# Uptake of antiretroviral therapy in HIV-positive women ever enrolled into ‘prevention of mother to child transmission’ programme, Mandalay, Myanmar—a cohort study

**DOI:** 10.1186/s12884-018-2099-0

**Published:** 2018-12-04

**Authors:** Khine Wut Yee Kyaw, Srinath Satyanarayana, Khaing Hnin Phyo, Nang Thu Thu Kyaw, Aye Aye Mon, Than Than Lwin, Thet Ko Aung, Myo Minn Oo, Zaw Zaw Aung, Thurain Htun, Nang Seng Noon Kham, Theingi Mya, Ajay M. V. Kumar, Htun Nyunt Oo

**Affiliations:** 1 Department of Operational Research, International Union Against Tuberculosis and Lung Disease (The Union), Mandalay, Myanmar; 2 Center for Operational Research, International Union Against Tuberculosis and Lung disease (The Union), Paris, France; 3 HIV unit, International Union Against Tuberculosis and Lung Disease (The Union), Mandalay, Myanmar; 4 National AIDS Programme, Department of Public Health, Ministry of Health and Sports, Nay Pyi Taw, Myanmar; 5 Monitoring, Evaluation, Accountability and Learning Unit, HIV, International Union Against Tuberculosis and Lung Disease (The Union), Mandalay, Myanmar; 6 Department of Obstetrics and Gynecology, Central Women Hospital, Mandalay, Myanmar; 7 Department of Operational Research, International Union Against Tuberculosis and Lung Disease (The Union), Delhi, India; 8 Department of Operational Research, International Union Against Tuberculosis and Lung Disease (The Union), Mandalay, Myanmar

**Keywords:** PMTCT, Myanmar, Operational research, ART, pARV

## Abstract

**Background:**

Early initiation and longer duration of anti-retroviral therapy either as prophylaxis (pARV) or lifelong treatment (ART) in HIV-positive pregnant women prior to delivery has a huge impact in reducing mother to child transmission (MTCT) of HIV, maternal morbidity, mortality and increasing retention in care. In this study, we aimed to determine the following in a ‘prevention of mother-to-child transmission’ (PMTCT) programme in Central Women Hospital, Mandalay, Myanmar: i) uptake of ART and factors associated with the uptake ii) duration of ART/ pARV received by HIV-positive pregnant women prior to delivery, iii) factors associated with ART/ pARV initiation after delivery and iv) factors associated with shorter duration of ART/ pARV (≤ 8 weeks prior to delivery).

**Method:**

This was a retrospective cohort study using routinely collected data from PMTCT programme. We used multivariable Cox proportional Hazard model or log binomial models to assess the association between socio-demographic and clinical factors with a) uptake of ART/pARV, b) initiation of ART/pARV after delivery, c) shorter (≤8 weeks) duration of ART/PARV prior to delivery.

**Results:**

Of the 670 ART naïve HIV-positive women enrolled to PMTCT programme between March 2011 and December 2016, 588 (88%) were initiated on ART/pARV. In adjusted analysis, only pregnancy stage at enrolment was significantly associated with initiation of ART/pARV. Of 585 who had delivered babies on or before the censor date, 522 (89%) were on ART/pARV. Women who lived outside Mandalay were more likely to be initiated on ART after delivery (i.e., delayed ART initiation in those on ART). Among women who were initiated on ART/pARV before delivery (*n* = 468), only 59% got ART/pARV for > 8 weeks before delivery. Women whose spouses’ HIV status was not recorded had 40% higher risk of short duration of ART/pARV.

**Conclusions:**

This study shows high uptake of ART/pARV among those enrolled into the PMTCT programme. However, about one in eight pregnant women did not receive ART before delivery. Among those initiated on ART/pARV before delivery, nearly half of them received ART/pARV for less than 8 weeks prior to delivery. These aspects need to be improved in order to eliminate mother-to-child transmission of HIV.

## Background

In pregnant women infected with human immunodeficiency virus (HIV), early diagnosis and antiretroviral therapy either as prophylaxis (pARV) or as lifelong treatment (ART) has several benefits such as prevention of opportunistic infections, reduction of morbidity and mortality and high retention on ART care in the long run in addition to reducing mother-to-child transmission (MTCT) of HIV [1–3]. Therefore, World Health Organization (WHO) recommends provider-initiated counselling and HIV testing to pregnant women attending antenatal care clinics in low-HIV prevalence setting as a key component of elimination of MTCT [4]. In addition to antenatal care, in high prevalence settings, provider-initiated counselling and HIV testing is also recommended at childbirth, postpartum and pediatric care setting to diagnose patients that may have been missed earlier or to identify women who have acquired new infections during this time period [4].

In Myanmar, HIV prevalence among adult population aged ≥15 years was estimated at 0.59% in 2015, a substantial decline compared to 0.94% in 2000 [5, 6]. UNAIDS estimated that there were 5100 pregnant women living with HIV in 2015 in Myanmar [7]. Myanmar has also set a target of reducing MTCT to < 5% by 2020 from the baseline of 15% in 2015 [8] and eliminating mother-to-child transmission of HIV by 2025. In order to achieve this, the National AIDS Programme has set 90–90-90 targets for HIV testing of pregnant women, provision of ART to HIV-positive pregnant women and provision of anti-retroviral prophylaxis to exposed babies [8]. The gaps in the provision of ART to HIV-positive pregnant mothers and antiretroviral (ARV) prophylaxis to exposed babies are largely unknown. HIV prevalence among pregnant women in Mandalay region of Myanmar was estimated at 0.81, 0.89 and 0.69% with testing rate of 41.4, 40.5 and 70.5% in 2012, 2013 and 2015 respectively [9].

In HIV-positive women who enrolled into ‘Prevention of mother-to-child transmission’ (PMTCT) programme in Central Women Hospital, Mandalay, the MTCT transmission rate was 2% [10]. However, the rates of transmission among HIV-positive pregnant women who failed to get enrolled into PMTCT services are unknown. In addition to early ART initiation, previous studies have shown that longer duration of ART can suppresses maternal viral load and each additional week of ART during antenatal period can reduce MTCT of HIV by 20% [11–13]. In Mandalay region, information on some of these programmatically relevant issues such as ART initiation, duration of ART before delivery, the factors associated with shorter duration of ART in HIV-positive pregnant women have not yet been studied and this information can help in improving the performance of the PMTCT programme.

In this study, we aimed to determine the following in pregnant women enrolled into the PMTCT programme in Mandalay, Myanmar: a) uptake of ART/pARV and factors associated with the uptake; b) duration of ART/pARV received by HIV-positive pregnant women prior to delivery; c) factors associated with ART initiation after delivery and d) factors associated with shorter duration of ART/pARV (≤ 8 weeks prior to delivery).

## Methods

### Study design

This was a retrospective cohort study involving secondary analysis of routinely collected data as part of the PMTCT programme of Central Women Hospital, Mandalay.

### Setting

Myanmar is one of the South-East Asian countries with a population of 51 million (in 2014) with 30% of population living in urban areas [14]. Mandalay region has 3rd largest population in the country (6.2 million) where the study was conducted [14].

### Prevention of mother to child transmission programme in central women hospital

Central Women Hospital is a 500-bedded public hospital providing maternal and child health care services in Mandalay. The International Union Against Tuberculosis and Lung Disease (The Union), an international non-governmental organization, has been implementing a PMTCT programme in Central Women Hospital in Mandalay in collaboration with National AIDS Programme (NAP), public hospitals and clinics under the Ministry of Health and Sports (MoHS) since 2011. The pregnant women attending antenatal care clinics at the public hospitals (central women hospital or township health departments) are offered HIV testing and referred to PMTCT clinic if they are tested HIV-positive. HIV testing at antenatal clinics is performed using a rapid, point-of-care, finger-prick test and is offered free of charge. HIV-positive pregnant women and post-partum women along with their exposed babies are referred and enrolled into PMTCT programme. Comprehensive PMTCT services are provided by a team of obstetricians and neonatologists from the Central Women Hospital, physicians from the Mandalay General Hospital, medical officers employed by The Union and People Living with HIV (PLHIV) network. HIV-positive women who were eligible for treatment were offered ART for life if their CD4 count was lower than cutoff point (350 cells/mm^3^ before 2015, 500 cells/mm^3^ from 2015 to 2016) and regardless of CD4 count after 2016. Women with CD4 count higher than cutoff point were given pARV as per the protocols of different time periods:

1. PMTCT option A (prior to 2013): women received Zidovudine (AZT) only.
2. PMTCT option B (from 2011 to 2014): women received triple ART during pregnancy, delivery and discontinued one week after breast feeding was stopped.
3. PMTCT option B+ (2014 to 2016): women received triple ART during pregnancy, delivery and continued for life [15, 16].

The management of HIV-positive women during post-partum period and exposed infants at central women hospital are described in detail elsewhere [10]. In brief, after delivery, mother and infant are followed-up until 18 months post-partum. Hence the follow up visit schedule depends on the infant feeding practices, the health status of mother and infant, availability of the attending physician and distance of patients’ residence from the hospital. The infants are tested for HIV, first at the age of 4–6 weeks and then at the age of 9 months. Infants tested positive for HIV antibodies are confirmed by another test between 9 and 18 months of age. All HIV-positive infants are transferred out to nearest pediatric integrated HIV Care clinic for ART initiation and continuation of care. All PMTCT services provided by the hospital are free of charge to the pregnant women and exposed infants.

### Study population

The study population includes ART-naïve HIV-positive women enrolled in Central Women Hospital’s PMTCT programme between March 2011 and December 2016. The HIV-positive women who were already on ART at the time of enrolment to PMTCT programme were excluded from the study analysis.

### Sources of data, data variables

Data of each study participant are collected in a structured proforma and entered into an electronic database of PMTCT programme routinely. We extracted data on the following variables: age, occupation, literacy, spouse’s HIV status, patient’s resident township, baseline CD4 count, baseline WHO clinical staging, baseline haemoglobin level**,** Hepatitis B, Hepatitis C, history of previous ART before enrolment, date of HIV diagnosis, date of ART initiation, ART regimen, and date of delivery.

### Statistical analysis

The data from the electronic database of the PMTCT programme was extracted and imported into STATA version 14.2 (College Station, TX). The uptake of ART and delivery status was assessed as of 31st March 2017 (censor date) or outcome date whichever date was earlier. To study the socio-demographic factors associated with uptake of ART/pARV, we used Cox proportional Hazards model. Hazards ratios (HR) and 95% confidence intervals (CI) were calculated. Among those initiated on ART/pARV, we used Log-binomial model to assess the factors associated with a) shorter duration of ART/pARV (duration of ART/pARV ≤8 weeks among who initiated on ART/pARV before delivery) and b) ART/pARV after delivery among HIV-positive mothers who had delivery record. A *P*-value of less than 0.05 was considered statistically significant for all analyses. The pregnancy stage at enrolment into PMTCT programme was categorized into before delivery, during delivery and post-partum periods.

## Results

**Fig. 1:**
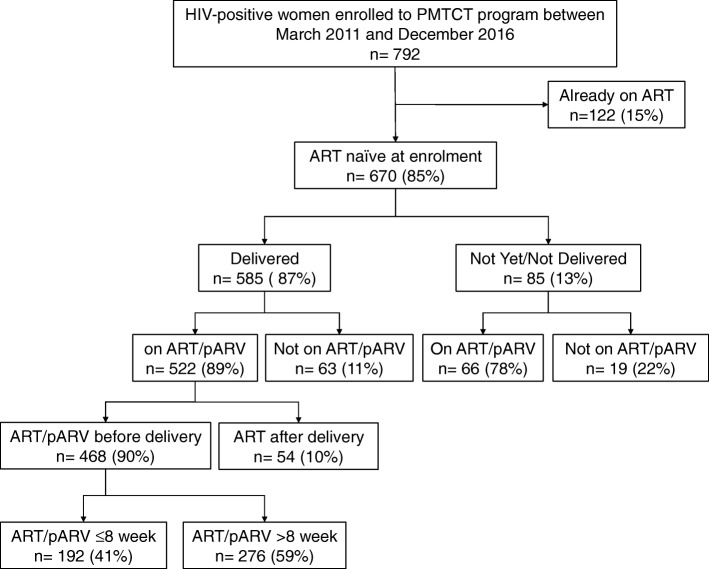
Flowchart of HIV-positive women enrolled in PMTCT programme in Central Women Hospital, Mandalay, Myanmar. The Fig. 1 described the flow of HIV positive women enrolled in PMTCT programme in Central Women Hospital, Mandalay, Myanmar. Footnote to Fig. 1: ^HIV^ Human Immunodeficiency virus, ^pmtct^ Prevention of mother to child transmission of HIV, ^ART^ Anti-retroviral therapy, ^pARV^ Anti-retroviral prophylaxis

There were 792 HIV-positive pregnant women enrolled into PMTCT programme between March 2011 and December 2016. The median age (interquartile range - IQR) and median CD4 cell count (IQR) at enrolment were 29 (25–33) years and 357 (218–511) cells/mm^3^ respectively. Among them, 122 (15%) had been initiated on ART before enrolment to PMTCT programme and were excluded for further analysis. The flowchart of HIV-positive women in PMTCT programme with ART status is described in Fig. 1.

Of 670 ART naïve HIV-positive pregnant women enrolled into PMTCT programme, 588 (88%) were initiated on ART/pARV and 82 (12%) were not initiated on ART/pARV. The uptake of ART under option A or option B was 42% and under option B+ was 58%. Among 588 women, 234 (39%) were initiated ART/pARV on the first day of enrollment and 433 (74%) were initiated within two weeks of enrolment into the PMTCT programme. Among those who were initiated on ART/pARV, the median (IQR) duration between enrolment into PMTCT programme and ART/pARV initiation was 7 (0–21) days and 485/588 (82%) were initiated on triple ART (option B or B+). Half (41 out of 82) of the women who were not initiated ART/pARV were lost to follow up (missed appointment more than three months from the last appointment day).

In unadjusted analysis, women whose spouse’s HIV status was recorded (either positive or negative), women with hemoglobin < 11 g% at enrollment and women who were enrolled in the programme during antenatal period were more likely to have been initiated on ART/pARV as shown in Table 1. We included factors with *p* value less than 0.15 in unadjusted analysis (spouse HIV status, baseline CD4 count, baseline hemoglobin level, literacy status and pregnancy stage at enrolment) in the multivariable model to obtain the adjusted hazard ratios. After adjustment, only ‘pregnancy stage at enrolment’ was significantly associated with initiation of ART [adjusted HR 4.4 (95% CI 3.2–6.1)] as shown in Table 1. The occupation, hepatitis B and Hepatitis C status were not significantly associated with initiation of ART (data not shown in Table 1).

**Table 1:** Characteristics and factors associated with initiation of ART/pARV in Central Women Hospital, Mandalay, Myanmar

Characteristics	Total n	ART or pARV initiation, *n* (%)	ART initiation rate (100 person-month-follow-up)	cHR (95% CI)	*P* value	aHR* (95% CI)	*P* value
Total	670	588 (88)	54				
Age (in years)							
<=30	400	348 (87)	48	1			
> 30	270	240 (89)	63	1.1 (0.9–1.3)	0.27		
Patients’ residence							
Outside MDY	159	139 (87)	61	1 (0.8–1.1)	0.55		
MDY	511	449 (88)	51	1			
Spouse HIV status recorded							
No	223	177 (79)	34	1		1	
Yes	447	411 (92)	72	1.3 (1.1–1.6)^§^	< 0.01	1.1 (0.9–1.4)	0.18
Baseline CD4 count (cells/mm3)							
<=350	311	297 (95)	93	1.2 (1–1.4)	0.06	1.1 (0.9–1.3)	0.28
> 350	302	271 (90)	42	1		1	
missing	57	20 (35)					
Baseline WHO staging							
I & II	311	272 (87)	50	1			
III & IV	359	316 (88)	57	1 (0.8–1.2)	0.75		
Baseline Haemoglobin level							
< 11 g%	372	357 (96)	97	1.2 (1.0–1.5)^§^	< 0.05	1.0 (0.8–1.2)	0.95
≥11 g%	234	205 (88)	36	1		1	
missing	64	26 (41)					
Literacy status							
Literate	622	553 (89)	53	1.5 (1–2.2)	0.05	1.4 (0.9–2.2)	0.11
Illiterate	36	25 (69)	44	1		1	
Missing	12	10 (83)					
Pregnancy stage at enrolment							
Before delivery	566	539 (95)	102	4.7 (3.5–6.4)^§^	< 0.001	4.4 (3.2–6.1)^§^	< 0.001
During deivery/Post-partum	104	49 (47)	9	1		1	

*ART* Anti-retroviral therapy, *pARV* Anti-retroviral prophylaxis, *cHR* crude Hazard Ratio, *aHR* Adjusted Hazard Ratio, *p* p value, *95% CI* 95% confident interval, *MDY* Mandalay

^§^Statistically significant

*Characteristics with *p* < 0.15 in unadjusted analysis were included in adjusted analysis

Of 670 HIV-positive women, 585 (87%) women had delivered their babies before censor date. Of the 85/670 (13%) who did not have a delivery record until censor date of 31st March 2017, 10/85 (12%) had died, 35/85 (41%) were lost to follow-up, 34/85 (40%) were transferred out to other ART centres before delivery and 6/85 (7%) were on regular follow-up. The median (IQR) follow up time for those 6 women on regular follow up was 20 (15–24) weeks.

Of 585 HIV-positive women who delivered babies, 522 (89%) were initiated on ART/pARV. Among them, 54/522(10%) were initiated on ART after delivery. Living outside Mandalay was significantly associated with initiation of ART after delivery with the adjusted risk ratio (95% CI) of 2.8 (1.7–4.6).

The duration of ART/pARV before delivery was calculated for 468 HIV-positive women who were initiated on ART/pARV before delivery and the median (IQR) duration of ART before delivery was 10 (5–14) weeks. About 192 (41%) women received ART/pARV for ≤8 weeks before delivery. Women whose spouse’s HIV status was unknown (i.e., not recorded) had 40% higher risk of shorter duration of ART [adjusted risk ratio (95% CI) 1.4 (1.2–1.8)].

Of the 585 children that were born, HIV test results were recorded for 410 children, and of those tested, 9 children were HIV positive (transmission rate of ~ 2.2%).

## Discussion

In this study, we found that 9 out of 10 women enrolled into the PMTCT Programme were initiated on ART/pARV with the rate of 54 per 100 person-months of follow-up. More than 70% were initiated on ART/pARV within 2 weeks of enrolment with 39% initiated on the date of enrollment to PMTCT programme. The proportion of HIV-positive women initiated on ART/pARV reported in our study was higher than global data on pregnant women living with HIV receiving medicines to prevent MTCT of HIV in 2016 (76%), in China (71%), in Cape town (46%), in Malawi where 63% in ART integrated model (HIV testing, ART provision integrated to antenatal care) and 51% in non-ART integrated model (only HIV testing integrated to antenatal care) [17–20].

We also found that women with anemia, women whose spouses had their HIV status ascertained and women who were enrolled into to PMTCT program before delivery were more likely to be initiated on ART/pARV. Anemia in HIV-positive pregnancy was usually associated with advanced HIV stage which might have led the clinician to initiate ART early for those with low hemoglobin levels [21]. Women whose spouses knew their HIV status might indicate better disclosure between the partners and better spouse support, which may be a facilitator for uptake of ART/pARV and longer duration of ART. This is similar to findings in other studies that show that involvement of spouse in care and disclosure of HIV status to partners enable initiation of ART, adherence to ART, and for minimizing fear, stigma and discrimination [22]. Our finding on lower rate of ART/pARV initiation among intrapartum/ post-partum women at enrolment may be due to the fact that clinician might give priority to pregnant women compared to those who have already delivered.

The major reason of non-ART/pARV was lost to follow-up and most of these happened within four weeks from enrolled date. This might be related to deficiencies in counselling on the importance of regular follow-up to clinic and importance of ART. In our setting, the counselling is being done predominantly to the pregnant women and this could be insufficient to bring them back to our clinic regularly as they may be dependent on other family members to come to the PMTCT clinic. In addition, there could be several other reasons for not initiating ART/pARV (as shown in other studies) such as patient’s refusal to get on to ART due to stigma, costs involved in accessing ART clinics (e.g., transportation cost), treatment seeking decision being made by husband, unwillingness to disclose HIV serostatus, long waiting time at the clinic and patient-unfriendly health care worker attitudes [22–24]. We did not explore these reasons in our study and therefore this is a potential area for further research.

Among HIV-positive mothers who delivered the babies and initiated on ART/pARV, 10% were initiated on ART after delivery and this proportion was relatively lower than a study conducted in Cape Town [2]. The delay in initiating ART was higher among HIV-positive women who lived outside Mandalay in our study. Patients living outside Mandalay might face several challenges in reaching our hospital such as financial constraints, inadequate transportation facilities, transportation cost, fear of stigma or discrimination and inadequate family support, similar to the reasons reported in other studies [22, 24, 25].

WHO recommends early ART in HIV-positive women (as soon as possible) well before delivery to be more effective in reducing mother to child transmission of HIV and studies show that at least 4–13 weeks of ART is required to achieve viral suppression at the time of delivery [26, 27]. About 40% of the women, who were initiated on ART, in our study were on ART for less than 8 weeks prior to delivery. We did not measure viral loads at the time of delivery and therefore what proportion of women in our study had low/suppressed viral loads at the time of delivery is unknown.

### Strengths

This is the first study conducted in Myanmar on the ART/pARV initiation and the delays involved in HIV-positive women enrolled under PMTCT programme. We used data that is collected by this programme under routine conditions and therefore this is likely to reflect ground realities. The findings of the study therefore have direct relevance to the hospital based PMTCT care setting. In addition, there is a system of routine data quality assurance in our programme and the data quality is regularly checked and corrected. Therefore, data errors, if any are likely to be minimal.

### Limitations

First, The PMTCT programme does not collect data on variables such as socio-economic status, last menstrual period or gestational week at enrolment and other factors shown to be associated in other studies [2, 22, 26]. Therefore, we were unable to study the association between these factors and ART initiation. Second, there was some missing data and we have tried to address this issue by creating a category for missing values and by not excluding such cases. We are not sure how this has affected the estimates used to study the associations in our study.

### Recommendations for strengthening the PMTCT programme and future research

First, an assessment should be conducted to know what proportion of eligible pregnant women are enrolled into PMTCT programme, the timing of their first antenatal care visit and time taken to enroll HIV-positive pregnant women into the PMTCT programme from the date of this first antenatal care visit. Second, qualitative studies to assess health seeking behavior among pregnant women, the reasons of late antenatal care presentation, delays in enrolment to PMTCT programme and barriers in ART initiation are required.

Third, recording and reporting of programme data should be strengthened and integrated with the hospital data to get full information on some important variables such as gestational week at HIV diagnosis and at enrolment to PMTCT programme, presence or absence of co-morbidities such as TB. Fourth, mechanisms to expand PMTCT care services to the decentralized ART centers should be strengthened so that the services are more accessible to all HIV-positive women (living outside Mandalay) and can result in earlier initiation of ART.

Fifth, lost to follow-up tracing should be strengthened and reasons for lost to follow-up must be periodically assessed and addressed. Lastly, effort must be made to increase spouse HIV testing and family support to HIV-positive pregnant women by raising awareness about it during antenatal care, PMTCT clinic and in community.

## Conclusion

This study shows high uptake of ART/pARV among HIV-positive women enrolled into the PMTCT programme in Myanmar. However, about 13% did not receive ART before delivery. Among those initiated on ART/pARV before delivery, nearly half of them did not receive ART/pARV for more than 8 weeks prior to delivery. These aspects need to be improved, if we are to eliminate mother to child transmission of HIV in Myanmar.

## Abbreviations

AIDS: Acquired Immune Deficiency Syndrome
aRR: Adjusted Risk Ratio
ART: Antiretroviral therapy
ARV: Antiretroviral drugs
CI: Confidence interval
HIV: Human Immunodeficiency Virus
HR: Hazard Ratio
IQR: Interquartile range
MDY: Mandalay
MTCT: Mother to child transmission
NAP: National AIDS Programme
PMTCT: Prevention of mother to child transmission
RR: Risk Ratio
UNAIDS: Joint United Nations Programme on HIV/AIDS
WHO: World Health Organization
